# Identification of Diarrheagenic *Escherichia coli* Strains from Avian Organic Fertilizers

**DOI:** 10.3390/ijerph110908924

**Published:** 2014-08-28

**Authors:** Juan Puño-Sarmiento, Luis Eduardo Gazal, Leonardo P. Medeiros, Erick K. Nishio, Renata K. T. Kobayashi, Gerson Nakazato

**Affiliations:** Department of Microbiology, Center of Biological Sciences, University of Londrina, Londrina, Paraná CP 6001, Brazil; E-Mails: jjoshua193@hotmail.com (J.P.-S.); gazal_26@hotmail.com (L.E.G.); leomedeiros27@gmail.com (L.P.M.); erick.nishio@yahoo.com (E.K.N.); kobayashirkt@uel.br (R.K.T.K.)

**Keywords:** *Escherichia coli*, diarrheagenic, organic fertilizer, avian, zoonotic risk, composting

## Abstract

The Brazilian poultry industry generates large amounts of organic waste, such as chicken litter, which is often used in agriculture. Among the bacteria present in organic fertilizer are members of the Enterobacteriaceae family. The objective of this study was to detect the presence of diarrheagenic *Escherichia coli* (DEC) strains in avian organic fertilizer, and assess the potential damage they can cause in humans due to antimicrobial resistance. The presence of DEC pathotypes and phylogenetic groups were detected by multiplex-PCR. Phenotypic assays, such as tests for adhesion, cytotoxicity activity, biofilm formation and especially antimicrobial susceptibility, were performed. Fifteen DEC strains from 64 *E. coli* were isolated. Among these, four strains were classified as enteropathogenic (EPEC; 6.2%), three strains as Shiga toxin-producing (STEC; 4.7%), 10 strains as enteroaggregative (EAEC; 12.5%), but two of these harbored the *eae*A gene too. The low number of isolated strains was most likely due to the composting process, which reduces the number of microorganisms. These strains were able to adhere to HEp-2 and HeLa cells and produce Shiga-toxins and biofilms; in addition, some of the strains showed antimicrobial resistance, which indicates a risk of the transfer of resistance genes to human *E. coli*. These results showed that DEC strains isolated from avian organic fertilizers can cause human infections.

## 1. Introduction

The use of improperly composted manure as organic fertilizer on farms can cause microbiological foodborne illnesses associated with the consumption of fresh fruits or vegetables [1,2,3]. Therefore, it is necessary to assess this risk factor to identify the possible effects of contamination in production areas.

Composting is a controlled, microbial process that converts biodegradable, organic materials into a stable, humus-like product. The main use of chicken litter is its application as soil fertilizer [4]. Due to its high concentration of nutrients and organic matter and the constant deposition of feces by poultry, this litter is a good substrate for the maintenance and development of a high and diverse microbial population. Some of these microorganisms are a major concern for public health and environmental quality because they can contaminate soil and water sources and infect humans and other animals via skin contact and the consumption of contaminated food or water [5].

Among the principal pathogenic microorganisms found in chicken litter are *Listeria monocytogenes*, *Mycobacterium avium*, *Candida albicans*, *Aspergillus fumigatus*, *Clostridium botulinum* and several serotypes of *Salmonella enterica* and *Escherichia coli* [6]. Miller and collaborators [7] showed that 30% of organic manure samples contain *E. coli*, indicating that under optimal conditions, the composting process may not be adequate to eliminate all pathogenic bacteria.

The compost is treated with high temperature to reduce the occurrence of pathogens from animal waste products. However, this process may not reduce pathogens to acceptable levels, or pathogens may be reintroduced into the finished product by cross-contamination on the farm. Pietronave and collaborators [8] showed that *Salmonella* and *E. coli* grew and survived longer in compost with a moisture content favoring these bacteria. *E. coli*, *Salmonella*, *E. coli* O157:H7 and *L. monocytogenes* had greater growth potential in sterilized compost compared to non-sterilized compost [9,10].

*E. coli* is a major commensal inhabitant of the intestine of humans and warm-blooded animals and is part of the essential microbiota that maintains the physiology of healthy hosts [11]. Occasionally, *E. coli* can cause several enteric and extra-intestinal diseases [12]. Such organisms associated with diarrhea are known as diarrheagenic *E. coli* (DEC) based on virulence markers, the pattern of adhesion to HEp-2 cells and clinical symptoms. Six different categories of DEC have been reported [12,13]: enteropathogenic (EPEC), enterotoxigenic (ETEC), enteroinvasive (EIEC), enterohemorrhagic (EHEC), enteroaggregative (EAEC) and diffusely adhering *E. coli* (DAEC).

There is concern about increased antimicrobial resistance in chicken litter because antimicrobials are used in food animal production to prevent disease or as growth promoters [14,15]. This resistance can be transferred to pathogenic strains through horizontal transfer in the gut of broilers and in manure and composted waste [16,17]. Thus, besides being pathogenic, a strain can present antimicrobial resistance, representing a danger to humans due to treatment complications.

The intention of this article is to demonstrate the presence of DEC in organic fertilizer samples collected from farms and to determinate the prevalence of antimicrobial resistance among the isolates that could represent a potential health risk for humans and animals.

## 2. Material and Methods

### 2.1. Samples and Bacterial Strains

A total of 40 avian organic fertilizer samples from 12 farmers were collected from the northern state of Paraná, Brazil, from December 2011 to June 2012. The fertilizer samples were collected from farmers outside of city of Londrina and one farmer at the State University of Londrina (UEL).

Approximately 25 g of fertilizer was gathered using collection jars and transported immediately to the laboratory. These samples were mixed in sterile water and diluted four times; the samples were spread on MacConkey agar (Difco^®^, Sparks, MD, USA) plates and incubated at 37 °C for 24 h. Two to three colonies from each plate were selected and examined using biochemical assays such as EPM, MILi and CITRATE [18,19,20], to confirm the presence of *E. coli*. Approximately 64 *E. coli* isolates were examined in this study. The samples were stored in Brain Heart Infusion (BHI) (Difco^®^) plus 25% glycerol (Sigma^®^, St. Louis, MO, USA) media at ‒80 °C. As positive and negative controls for the PCR, adherence and biofilm assays, the following reference strains were used: *E. coli* E2348/69 (O127:H6, *eae*+, *bfp*A+, localized adherence), *E. coli* EDL 933 (O157:H7, *eae*+, *stx*1+, *stx*2+, *ehx*A+), EAEC O42 (*agg*R+, aggregative adherence), EIEC EDL 1284 (*ipa*H+), ETEC H10407 (*elt*+), ETEC B41 (*est*+), *E. coli* J96, *E. coli* K12Hb101 (negative control), and *E. coli* strain 20 [21] (Bacterial Collection of the Laboratory of Basic and Applied Bacteriology from State University of Londrina).

### 2.2. DNA Extraction

All strains were grown on TSA agar (Difco^®^) at 37 °C for 24 h. DNA was extracted by suspending seven bacterial colonies in 200 µL of sterile water; the sample was heated at 100 °C for 10 min and centrifuged at 10,000 × *g* for 6 min. The supernatant was used as the template in PCR assays.

### 2.3. Detecting virulence genes using polymerase chain reaction (PCR)

The presence of virulence genes was examined using two multiplex PCR techniques. The following virulence markers were used to detect DEC: *eae* (structural gene for intimin of EPEC and EHEC), *bfp*A (structural gene for the BFP of typical EPEC), *agg*R (transcriptional activator for the AAFs of EAEC), *elt*, *est* (enterotoxins of ETEC), *ipa*H (invasion plasmid antigen H found in EIEC *Shigella*), *stx*1, *stx*2 (Shiga toxins of EHEC) and *ehx*A (enterohemolysin, which can be found in EHEC). Multiplex PCR test methods described previously [22,23] were used, with slight modifications. For each gene, the primers sequence, the size of the DNA fragment produced, and the concentration of each primer used in the reaction are described in Table 1. The amplification of bacterial DNA was performed using 2.5 µL of DNA template in 25 µL of reaction mixture containing 1.5 U *Taq* polymerase (Invitrogen^®^, Carlsbad, CA, USA), 0.2 mM of each deoxynucleoside triphosphate (dNTP, Invitrogen^®^), 2.5 mM MgCl_2_, 2.5 µL PCR Buffer 10× (Invitrogen^®^), the appropriate primers (Table 1) and sterile water. The PCR was carried out programming a thermal cycler (Veriti^®^ Applied Biosystems^®^, Foster City, CA, USA) with an initial denaturing cycle of 95 °C for 5 min and 40 cycles of 95 °C for 1 min, 54 °C for 1 min and a final cycle of 72 °C for 1 min, as used by Aranda and collaborators [22]. For the multiplex PCR described by Paton and Paton [23], samples were subjected to 5 min of denaturing at 95 °C, 15 cycles of 94 °C for 1 min, 65 °C for 2 min, 72 °C for 1.5 min and 20 cycles of 94 °C for 1 min, 60 °C for 2 min, 72 °C for 2.5 min and a final cycle of elongation at 72 °C for 7 min. A 10-µL aliquot of each reaction mixture was subjected to electrophoresis on 2% agarose gel, followed by staining with 1 µL Gel Red 20× (Biotium^®^, Hayward, CA, USA) and visualization using a UV transilluminator. A 1-kb DNA ladder (Invitrogen^®^) was loaded on each gel.

**Table 1:** PCR primers used in this study.

Gene	PCR primer	Primer sequence (5’–3’)	Fragment (bp)	Concentration (pmol/µL)	ControlStrains	Reference
Primers used for multiplex PCR described by Aranda and collaborators [22]						
*eae*A	F	CTGAACGGCGATTACGCGAA	917	10	E2348/69	[24]
	R	CCAGACGATACGATCCAG			(EPEC)	
*bfp*A	F	AATGGTGCTTGCGCTTGCTGC	326	1.25	E2348/69	[24]
	R	GCCGCTTTATCCAACCTGGTA			(EPEC)	
*agg*R	F	GTATACACAAAAGAAGGAAGC	254	2.5	O42	[25]
	R	ACAGAATCGTCAGCATCAGC			(EAEC)	
*elt*	F	GGCGACAGATTATACCGTGC	450	0.25	H10407	[24]
	R	CGGTCTCTATATTCCCTGTT			(ETEC)	
*est*	F	ATTTTTMTTTCTGTATTRTCTT	190	6.47	B41	[24]
	R	CACCCGGTACARGCAGGATT			(ETEC)	
*ipa*H	F	GTTCCTTGACCGCCTTTCCGATACCGTC	600	1	EDL 1284	[24]
	R	GCCGGTCAGCCACCCTCTGAGAGTAC			(EIEC)	
*stx*	F	GAGCGAAATAATTTATATGTG	518	6	EDL 933	[26]
	R	TGATGATGGCAATTCAGTAT			(EHEC)	
Primers used for multiplex PCR described by Paton and Paton [23]						
*eae*A	F	GACCCGGCACAAGCATAAGC	384	10	E2348	[23]
	R	CCACCTGCAGCAACAAGAGG			(EPEC)	
*stx1*	F	ATAAATCGCCATTCGTTGACTAC	180	10	EDL 933	[23]
	R	AGAACGCCCACTGAGATCATC			(EHEC)	
*stx2*	F	GGCACTGTCTGAAACTGCTCC	255	10	EDL 933	[23]
	R	TCGCCAGTTATCTGACATTCTG			(EHEC)	
*ehxA*	F	GCATCATCAAGCGTAGCTTCC	534	10	EDL 933	[23]
	R	AATGAGCCAAGCTGGTTAAGCT			(EHEC)	
Primers used for multiplex PCR described by Clermont and collaborators [27]						
*chuA*	F	GACGAACCAACGGTCAGGAT	279	20	*E. coli*	[27]
	R	TGCCGCCAGTACAAAGACA			J96	
*yjaA*	F	TGAAGTGTCAGAGACGCTG	211	20	*E. coli*	[27]
	R	ATGGAGAATGCGTTCCTCAAC			J96	
*TspE4C2*	F	GAGTAATGTCGGGGCATTCA	152	20	*E. coli*	[27]
	R	CGCGCCAACAAAGTATTACG			Strain 20	

### 2.4. Determination of Phylogenetic Group (PCR)

*E. coli* strains were classified into phylogenetic groups using the PCR-based technique previously described by Clermont and collaborators [27]. For the PCR assay, the following genes were used: *chuA* (required for heme transport in enterohemorrhagic O157:H7 *E. coli*), *yjaA* (initially identified in the recent complete genome sequence of *E. coli* K-12) and a fragment of *TspE4C2* (anonymous fragment). The PCR test for phylogenetic group was performed using 2.5 µL of supernatant in 10 µL of reaction mixture containing 1.5 U Taq polymerase (Invitrogen^®^), 0.2 mM of each deoxynucleoside triphosphate (dNTP, Invitrogen^®^), 2.5 mM MgCl_2_, 2.5 µL PCR Buffer 10X (Invitrogen^®^) and the appropriate primers (Table 1). The following cycling parameters were used: 94 °C for 4 min and 30 cycles of 94 °C for 5 s, 59 °C for 10 s and a final cycle of 72 °C for 5 min. A 10-µL aliquot of each reaction mixture was subjected to electrophoresis on a 2% agarose gel, followed by staining with 1 µL Gel Red 20× (Biotium^®^) and visualization using a UV transilluminator. A 1-kb DNA ladder (Invitrogen^®^) was loaded on each gel.

### 2.5. Adherence Assays

*E. coli* adherence to HEp-2 and HeLa cells was detected as previously described [28], with slight modifications. Cells were grown in 24-well tissue culture microplates (BD Falcon, Bedford, MA, USA) in which sterile round cover slips (13 mm in diameter) were placed prior to inoculation. The growth medium in each well of the microplate consisted of 0.9 mL of Eagle’s minimal essential medium (MEM, Invitrogen^®^) supplemented with 10% fetal calf serum (Invitrogen^®^) and 1% antibiotic solution (penicillin 100,000 U and streptomycin 100 µg/mL, Sigma^®^). The HEp-2 monolayer was grown overnight at 37 °C with 5 % CO_2_ to yield at least 70% confluence. The slides were washed three times with sterile phosphate-buffered saline 0.05 M, pH 7.4 (PBS). Forty microliters of the overnight bacterial culture was incubated in Luria Bertani Broth (LB, Difco^®^) at 37 °C and added to 0.96 mL of MEM containing 2% fetal calf serum and 3% D-mannose (Sigma^®^). After 3 h of incubation at 37 °C with 5% CO_2_, the monolayers were washed with sterile PBS and incubated for an additional 3 h. Next, the slides were washed five times with PBS, fixed with absolute methanol for 10 min and stained with May-Grunwald and Giemsa stain. The slides were examined under a light microscope using an oil immersion lens. To determine the adhesion pattern, previously described criteria were used [29,30,31].

### 2.6. Antimicrobial Susceptibility Testing 

All *E. coli* isolates that exhibited some virulence factor were tested with antimicrobial agents on Mueller Hinton agar (Difco^®^) using the agar disk diffusion method, as recommended by the Clinical and Laboratory Standards Institute [32]. The following antimicrobial agents (Laborclin^®^, Pinhais, PR, BR) were used: amoxicillin (AC, 10 µg), amoxicillin-clavulanic acid (AMC, 30 µg), aztreonam (ATM, 30 µg), tetracycline (TET, 30 µg), cefotaxime (CTX, 30 µg), imipenem (IPM, 10 µg), nalidixic acid (NAL, 30 µg), gentamicin (GEN, 10 µg), chloramphenicol (CHL, 30 µg), trimethoprim-sulfamethoxazole (SXT, 25 µg), ampicillin (AMP, 10 µg), ciprofloxacin (CIP, 5 µg), norfloxacin (NOR, 10 µg) and streptomycin (STR, 10 µg). Enrofloxacin (EFX, 5 µg) was also tested because this antimicrobial is commonly used in veterinary clinics. *E. coli* strain ATCC 25922 was used as a quality control for the antimicrobial susceptibility testing.

### 2.7. Biofilm

Biofilm formation was assessed by the methodology described by Wakimoto and collaborators [33], with slight modifications. The strains were grown in Luria Bertani (LB) medium for 24 h at 37 °C with shaking. Then, 5 µL of culture was inoculated in 200 µL of Dulbecco’s modified Eagle medium (DMEM) containing 0.45% glucose in 96-well flat-bottom microliter polystyrene plates (BD Falcon). The plates were covered and incubated aerobically for 24 h at 37 °C. Before staining for five minutes with 0.5% crystal violet, the samples were washed four times with PBS 0.01 M, pH 7.4.The dye bound to adherent cells was solubilized with 200 µL of 95% (v/v) ethanol per well. The biofilm was quantified at 570 nm using an automated plate reader (Synergy™ HT, Bio-Tek, Winooski, VT, USA). Strain EAEC O42 and *E. coli* HB101 were used as a positive and negative control, respectively. The isolates were evaluated according to Wakimoto and collaborators into three categories: group 1 (OD_570_ > 0.2), strong biofilm formation; group 2 (0.1 ≤ OD_570_ ≤ 0.2), moderate biofilm formation and group 3 (OD_570_ < 0.1), without biofilm formation (Table 2).

### 2.8. Assay for Cytotoxicity Activity

The MTT [3-(4,5-dimethylthiazol-2-yl)-2,5-diphenyltetrazolium bromide] colorimetric assay was performed to quantify viable cells in a Vero cell culture after adding the supernatant of a potential toxic agent, Shiga toxin. This assay was based on the method of Murakami and collaborators [34]. Supernatants of STEC isolates, obtained by centrifugation and filtration, as previously described [35], were added to 96-well plates at a concentration of 2 × 10^5^ Vero cells per well in an end-volume of 100 µL containing MEM and supplemented with 2% fetal calf serum and 1% antibiotic solution (penicillin 100,000 U and streptomycin 100 µg/mL); the cells were incubated in 5 % CO_2_ for 72 h at 37 °C. The toxin dilution added was 1:10 and was performed in triplicate. The culture medium was removed, and the wells were washed with 200 µL of PBS 0.01 M. MTT stock solution (Sigma^®^) was added and incubated at 37 °C for 4 h in a 5 % CO_2_ atmosphere. The medium was removed, and 200 µL of lysis reagent was added. The amount of converted MTT solution was quantified using a spectrophotometer at 595 nm. The quantity of produced purple formazan was assumed to correlate with the amount of viable cells remaining.

### 2.9. Statistical Analysis 

Significant differences among the results were examined using *X*^2^ (Chi-square test) for bacterial groups; differences were considered significant at *p* < 0.05. The data were expressed as the means ± SD. The statistical analyses were performed using the BioEstat version 5.0 software (Instituto Mamiraúa, Belém, PA, BR).

## 3. Results

### 3.1. E. coli Isolates

A total of 40 organic fertilizer samples were collected in this study. A total of 64 *E. coli* colonies were isolated, and 15 strains (30%) were found to be positive for DEC. Some strains showed a variable biochemistry profile, including non-fermentative lactose such as OF-4 and lysine decarboxylase negative such as OF-57; other strains were also negative for motility: OF-8, OF-9, OF-6, and OF-57.

### 3.2. Virulence Factors of Diarrheagenic E. coli (DEC)

Of the 64 *E. coli* isolates, 15 (23.4%) strains were positive for the virulence factors of EPEC, STEC and EAEC (Table 2). These results showed that the organic fertilizer samples carry virulence factors.

The PCR assays detected four strains (6.2%) with the *eae*A gene (OF-4, OF-36, OF-42, OF-51). The *agg*R gene (EAEC) was predominant. Ten isolates (12.5%) presented this gene, but two of these also had the *eae*A gene (OF-42, OF-51), and the *stx*1 gene (STEC) was found in three strains (4.7%; *p* < 0.05) such as OF-22, OF-23 and OF-24.

### 3.3. Phylogenetic Group

The majority of the DEC strains belonged to phylogenetic group A (60%) and B1 (33.3%) and were genetically diverse. Only the strain OF-36 (atypical EPEC) was classified as a member of phylogenetic group B2 (Table 2), and therefore, the incidence of groups A and B1 was significantly higher than group B2 (*p* < 0.05).

### 3.4. Adherence to HEp-2 and HeLa Cells

**Figure 1:**
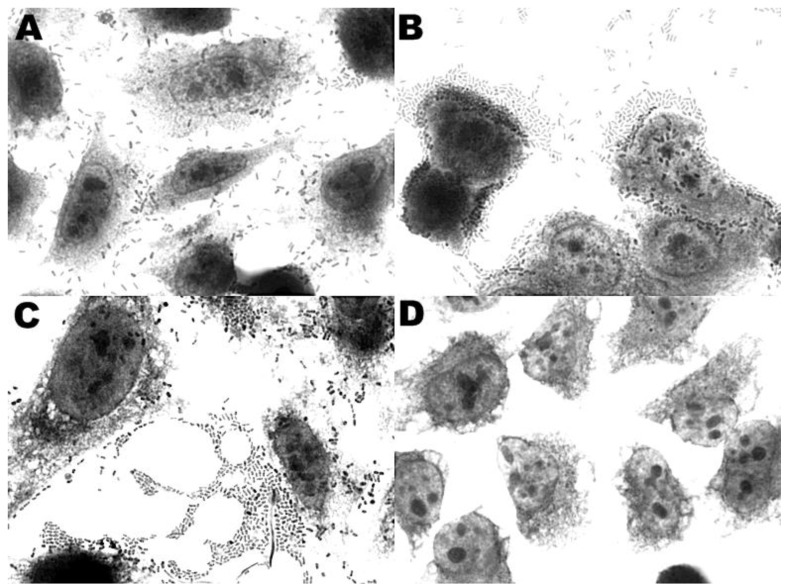
Adherence of *E. coli* isolated in this study (microscope magnification of 1000×): (**A**) and (**B**) OF-39 strain showing diffuse adherence and aggregative adherence on HEp-2 cells; (**C**) OF-9 EAEC strain showing aggregative adherence on HEp-2 cells and (**D**) K12 Hb101 negative control of adherence test.

The only adherence pattern observed in the DEC isolates when cultured with HEp-2 and HeLa cells was aggregative adherence (AA-100%) (Figure 1A) and was significantly predominant in the organic fertilizer samples (*p* < 0.05). Only isolate OF-34 (Table 2) showed a mixed adherence pattern, a combination of aggregative and diffuse adherence (Figure 1B). All of the isolates showed the same adherence pattern for both cell types.

### 3.5. Antimicrobial Susceptibility

Resistance to 15 antibiotics was examined in the *E. coli* isolates that showed some virulence factor (Table 2). Only two strains (OF-36 and OF-42) did not show any resistance to antibiotics compared to the 86% of isolates that were resistant to at least one antibiotic group (*p* < 0.05). Three isolates were resistant to more than three antibiotics, and isolate OF-61 showed resistance to seven antibiotics.

### 3.6. Biofilm Test

The isolates were classified into three groups based on absorbance: group 1 (OD_570_ > 0.2), 5 strains with strong biofilm formation (OF-4, OF-6, OF-8, OF-22, and OF-23); group 2 (0.1 ≤ OD_570_ ≤ 0.2), 1 strain (OF-35) considered as moderate and group 3 (OD_570_ < 0.1), 9 strains without biofilm formation (Table 2). The isolates that exhibited biofilm formation showed an aggregative adherence pattern.

### 3.7. Evaluation of Cytotoxic Activity

The MTT assay was positive for all of the STEC isolates. The three STEC strains showed cytotoxic activity, but *E. coli* OF-22 showed a toxigenic potential (80%) higher than the strains OF-23 and OF-24 (*p* < 0.05), at 61% and 52%, respectively. These results were visualized by a rounding of the cells under microscopy, indicating cell death and thus cytotoxic activity of the supernatant.

**Table 2:** Genotypic and phenotypic analysis of diarrheagenic *E. coli* isolates collected from organic fertilizer samples.

Isolate of *E. coli*	Genetic profile	Pattern of adherence in HEp-2 cell	Resistance profile	Biofilm formation (BF)	Phylogenetic group
OF-4	*eae*+	AA	NAL/AMO/SRT	Strong	A
OF-36	*eae*+	AA	Susceptible	Non-BF	B2
OF-42	*eae*+/*agg*R+	AA	Susceptible	Non-BF	A
OF-51	*eae*+/*agg*R+	AA	AMO ^a^/TET	Non-BF	B1
OF-6	*agg*R+	AA	AMO ^a^	Strong	A
OF-8	*agg*R+	AA	AMO ^a^/ACL	Strong	B1
OF-9	*agg*R+	AA	NAL ^a^/AMO ^a^/AMC^a^/IMP^a^/AMP	Non-BF	B1
OF-22	*stx*1+	AA	AMO ^a^/AMP ^a^	Strong	A
OF-23	*stx*1+	AA	NAL ^a^/AMO ^a^/AMP ^a^	Strong	B1
OF-24	*stx*1+	AA	AMO ^a^	Non-BF	A
OF-35	*agg*R+	AA	TET	Moderate	A
OF-39	*agg*R+	AA/DA	AMO ^a^/AMP ^a^	Non-BF	B1
OF-44	*agg*R+	AA	STR/SXT/TET	Non-BF	A
OF-57	*agg*R	AA	STR ^a^	Non-BF	A
OF-61	*agg*R	AA	NAL/AMO/AMC/AMP/STR/IMP/ TET	Non-BF	A

Notes: AA, aggregative adherence; DA, diffuse adherence; AMO, amoxicillin; AMC, amoxicillin-clavulanic acid; AMP, ampicillin; TET, tetracycline; IMP, imipenem; NAL, nalidixic acid; SXT, trimethoprim-sulfamethoxazole; STR, streptomycin. ^a^ Resistance to the indicated drug is intermediate or resistant according to CLSI standards.

## 4. Discussion

Most organic fertilizers analyzed in our study underwent some type of exothermic process under conditions of aeration and moisture for a sufficient time to inactivate food-borne pathogens. In Brazil, chicken litter is very useful as an organic fertilizer soil for the production of fruits and vegetables [5]. However, the composting process that is intended to reduce the presence of pathogens from animal waste products may not reduce them to acceptable levels on farms. Moreover, STEC and EHEC strains can persist in the environment for long periods of time, and the consumption of these pathotypes is associated with severe clinical illness in humans [36,37]. In an investigation of an outbreak of EHEC strain O157:H7 associated with apple cider in south-eastern Massachusetts, which presumably had been contaminated by manure used as fertilizer in the orchard, showed that the organism survived for 20 days at a pH value below 4, conditions considered sufficient to inhibit the growth and survival of bacterial pathogens [38].

In our study, we had difficulty in isolating *E. coli* and other species due to the composting process that was able to decrease the presence of these microorganisms. However, we found 15 DEC strains identified as EAEC, STEC and EPEC (Table 2), which are considered a potential risk of *E. coli* contamination in fresh produce. EAEC was the most commonly isolated pathotype in the organic fertilizer samples. EAEC is emerging as an enteric pathogen of great importance and has been associated with traveler’s diarrhea in both developing and industrialized countries [39]. The last outbreak occurred in Germany in 2011, with 3.368 cases, including 36 deaths (European Centre for Disease Prevention and Control), and was the second largest outbreak foodborne by *E. coli* in the history. This outbreak was caused by the EHEC strain O104:H4, which shows high genetic similarity with EAEC 55989 [40]. Despite the rapid identification and characterization of this strain, there was a great difficulty in identifying the source of contamination because many outbreaks of infection are associated with water, animals and food directly or indirectly contaminated with organic or inorganic fertilizers [41]. 

Four EPEC strains were found in this study, and this pathotype is highly linked to childhood diarrhea. The EPEC target is the small intestine and causes histopathological alterations termed “attaching and effacing (A/E)” lesions on the surface of intestinal epithelial cells; in particular, infants in developing countries are the most affected [42]. The ability to cause A/E lesions is encoded by a large bacterial chromosomal pathogenicity island, the locus of enterocyte effacement (LEE), which contains the gene *eae* that encodes intimin (94 to 97 kDa outer membrane protein) [43]. Moreover, there are reports of EPEC infection in animals, such as bovines [44], pigs [45], dogs and cats [46,47]. However, as there are few studies involving EPEC in broilers, this work provides interesting results about this issue.

Although EHEC is the pathotype most relevant to outbreaks of human infection, there are very few reports about STEC alone. In this work, we found strains harboring *stx*1 gene, responsible for cause severe diarrhea in humans which may result in complications such as hemolytic-uremic syndrome (SHU) [13]. Moreover, with a higher toxigenic potential, *E. coli* OF-22 showed an effect of up to 80% in endothelial cells (Vero cells).

We performed two multiplex PCR assays to identify the five principal pathotypes of DEC in organic fertilizer samples. These PCR assays have been validated in humans’ strains [22] and other animals, such as dogs and cats [47]. Both multiplex PCR assays showed a high specificity in identifying DEC from different sources therefore offering a practical possibility for identification in studies involving a large number of strains. In this work, the use of two methodologies to identify virulence genes was very important because one multiplex assays for the five pathotypes and the other assists in the identification and helps in the subtyping of the *stx* gene. We believe that these are complementary techniques.

The adherence to epithelial cells in culture is one of the most important tests for infection capability. All of the DEC strains isolated in this study showed aggregative adherence to human cells (HEp-2 cells), showing a danger of human infection. Furthermore, adherence assays confirmed PCR results for EAEC strains because the AA pattern is the only adherence pattern that was describe for this pathotype in humans and animals.

Although organic fertilizers from chicken litter are very useful for food production, they may have microorganisms carrying antimicrobial resistance genes that can be transferred to human pathogenic bacteria. This represents a risk for the public health, by complicating treatments with antibiotics [5,7].

In the poultry industry, antimicrobials are used therapeutically, non-therapeutically and also as growth promoters [14,48]. Thus, the use of antimicrobials in animal production may promote the dissemination of resistance genes beyond that expected as a direct consequence of selective pressure on target organisms [49]. These microorganisms can be introduced into the farm environment many times through animal feces [50], which could lead to the contamination of neighboring water sources and agricultural crops [51]. Sulfonamides, amoxicillin, tetracyclines, tylosin, neomycin and penicillin are used to treat bacterial diseases in the poultry industry [49]. We found ten isolates with resistance to amoxicillin, four isolates with resistance to tetracycline and one strain with resistance to sulfonamide, and reports confirm the prevalence of resistance to these antibiotics in broiler chickens and poultry manure [7,15,52,53]. Until 2005, quinolones were frequently used for the treatment of certain infections in poultry to control mortality, but quinolone-resistant *Campylobacter* strains began to emerge; thus, the Food and Drug Administration (FDA) banned the use of that drug in poultry [54]. Our results showed only one strain (OF-61; EAEC) with resistance to nalidixic acid, but this isolate presented resistance to six other groups of antimicrobials. This multi-resistant strain represents a public health hazard.

Phylogenetic grouping of *E. coli* is associated to virulence pathotypes [27,55]. *E. coli* strains can be divided in four main phylogenetic groups: A, B1, B2 and D [27], according to the presence or absence of *chu*A and *yja*A genes, and a fragment of *TspE4.C2*, later characterized as a lipase esterase gene [56]. The majority of our isolates of DEC belonged to groups A (60%) and B1 (33.3%). Similar results were reported by Escobar-Páramo and collaborators [57] in a phylogenetic analysis from different bacterial collections isolated from several studies, in which most groups into D and B2, with only one strain of EPEC classified as group B2. Another study performed by Miller and collaborators [7] showed the same results in obligatory pathogens responsible for acute and severe diarrhea. Our results showed the same groups A and B1 in *E. coli* strains isolated from organic fertilizers, with the same antibiotic resistance found by Miller and collaborators in their study.

To our knowledge, the present study is the first report to focus on the presence of five principal pathotypes of DEC in organic chicken fertilizer used on farms that could represent a significant public health safety hazard. Our findings suggest that some strains have developed multi-resistance to antibiotics.

## 5. Conclusions

This study shows that pathogens may be present in organic fertilizers after the thermophilic phase of composting and provides important data with regard to the epidemiological aspects of infections caused by *E. coli*. The presence of these DEC strains with resistance to antimicrobials represents a great risk to public health.
